# Preclinical Evaluation of the HDAC Inhibitor Chidamide in Transformed Follicular Lymphoma

**DOI:** 10.3389/fonc.2021.780118

**Published:** 2021-12-03

**Authors:** Mengya Zhong, Jinshui Tan, Guangchao Pan, Yuelong Jiang, Hui Zhou, Qian Lai, Qinwei Chen, Liyuan Fan, Manman Deng, Bing Xu, Jie Zha

**Affiliations:** ^1^ Department of Hematology, The First Affiliated Hospital of Xiamen University and Institute of Hematology, School of Medicine, Xiamen University, Xiamen, China; ^2^ Department of Hematology, Key Laboratory of Xiamen for Diagnosis and Treatment of Hematological Malignancy, Xiamen, China

**Keywords:** transformed-follicular lymphoma (t-FL), chidamide, HDAC, PI3K/AKT signaling, epigenetic antitumor therapy

## Abstract

The key factors leading to transformed follicular lymphoma (t-FL) include the aberrations of epigenetic modifiers as early and driving events, especially mutations in the gene encoding for histone acetyltransferase. Therefore, reversal of this phenomenon by histone deacetylase (HDAC) inhibitors is essential for the development of new treatment strategies in t-FL. Several t-FL cell lines were treated with various doses of chidamide and subjected to cell proliferation, apoptosis and cell cycle analyses with CCK-8 assay, Annexin V/PI assay and flow cytometry, respectively. Chidamide dose-dependently inhibited cell proliferation, caused G0/G1 cycle arrest and triggered apoptosis in t-FL cells. In addition, the effects of chidamide on tumor growth were evaluated *in vivo* in xenograft models. RNA-seq analysis revealed gene expression alterations involving the PI3K-AKT signaling pathway might account for the mechanism underlying the antitumor activity of chidamide as a single agent in t-FL. These findings provide a basis for further clinical exploration of chidamide as a promising treatment for FL.

## Introduction

Follicular lymphoma (FL) is the most common indolent lymphoma, accounting for approximately 20% of all non-Hodgkin lymphoma (NHL) cases (1, 2). With the use of current front-line regimens, the majority of FL patients have an initial response to therapy, with 40 to 80% demonstrating complete response (3, 4). However, despite improvement in front-line treatment, conventional therapy for FL is not curative, and approximately 20% of patients still experience either refractoriness or early relapse, which occurs in the first 2 years after diagnosis and treatment by chemoimmunotherapy (5–7). Moreover, such early relapse cases are often chemo-resistant, leading to significantly shorter survival (4, 8). Thus, integrating molecular targeted therapies into current treatment protocols and adjusting conventional treatment to improve survival, without compromising long-term quality of life is urgently needed in FL patients with poor prognosis.

Histone deacetylases (HDACs), which act as “epigenetic erasers”, are known to catalyze the removal of acetyl groups from histones and non-histone proteins, thereby altering the transcription of oncogenes and tumor suppressor genes (9, 10). Aberrant HDAC expression occurs in both solid tumors and hematological cancers, including B-cell lymphoma (11, 12). Prior findings showed that dysregulation of histone acetylation contributes to lymphomagenesis, particularly in GC-derived lymphomas (13, 14). FL, similar to other cancers, has recurrent alterations in genes involved in maintaining chromatin structure and transcription machinery genes (4, 8). For example, somatic mutations or genomic loss in the CREBBP and EP300 genes that encode HATs lead to an imbalance between acetylation and deacetylation, and the occurrence of these mutations is associated with disease relapse and poor prognosis in FL (15–17). Perturbing the balance between histone acetylation and deacetylation, which is tightly regulated by HDACs, is one of the main mechanisms by which epigenetics may be exploited to harness chromatin remodeling (18). In this context, it raises the possibility that HDACs may serve as a potentially attractive therapeutic target in this disease.

According to previous studies, Class I HDACs 1-3 are the most important HDAC enzymes with close associations with the corresponding malignant phenotypes (19, 20). Currently, multiple HDAC inhibitors have been developed, Chidamide is a noteworthy drug that may target specifically subtypes 1, 2 and 3 of Class I and subtype 10 of Class IIb HDACs and lead to increased acetylation of histones H3 and H4, resulting ultimately in the activation of gene transcription (21, 22). Chidamide was first approved by the Chinese FDA for the treatment of relapsed or refractory (R/R) peripheral T cell lymphoma (PTCL) (23, 24). Afterwards, more and more studies have focused on the anti-cancer effects of chidamide in various tumors, including hematological tumors (25–27). However, significant knowledge gaps remain, including the mechanism underlying chidamide’s therapeutic effects. In addition, its clinical utility in FL is currently unclear.

This study evaluated the effects of chidamide in well-characterized transformed follicular lymphoma cell lines and a xenograft model of t-FL. In addition, we analyzed the molecular basis of chidamide’s effects by evaluating gene expression using microarrays in cells treated with the single agent in t-FL: 1) to identify genes and pathways affected by chidamide; 2) to determine biomarkers that could be used in preclinical studies.

## Materials and Methods

### Cell Lines and Molecules

Established human cell lines derived from t-FL, including RL, DOHH2, SU-DHL4 and Karpas422 cells, were obtained from Cobioer Biotechnology Company (Jiangsu, China). All cell lines were cultured at 37°C in a 5% CO_2_ incubator in RPMI-1640 (Gibco, CA, USA) supplemented with 10% fetal bovine serum (FBS, Gibco, CA, USA), 100 units/ml penicillin and 100 mg/ml streptomycin (Invitrogen, CA, USA). Chidamide (CS055; HBI-8000) was provided by Shenzhen Chipscreen Biosciences (Shenzhen, China) and dissolved in sterile DMSO (Sigma, MO, USA) to produce a 50-mM stock solution stored at -20°C for *in vitro* experiments and diluted in 0.5% (w/v) CMC-Na suspension for oral gavage.

### Cell Viability Assessment

Cytotoxicity was determined with Cell Counting Kit-8 (CCK-8, APExBIO, Texas, USA). Briefly, 3×10^4^ cells/well were seeded in 100μl medium in 96-well plates and treated with various concentrations of chidamide alone for 24, 36 and 48 h. The CCK-8 reagent (10μl/well) was then added and incubated for additional 2 h, after which absorbance at 450 nm was detected on a Bio-Rad microplate reader (Bio-Rad, CA, USA). Data from three independent triplicate experiments were presented as a percentage of viable cells relative to untreated controls. IC_50_ values were determined with the GraphPad Prism 6 software.

### FACS Analysis of the Cell Cycle and Apoptosis

Cells were treated with various concentrations of chidamide for the designated times. Cells were harvested and processed according to the manufacturer’s protocols. For cell cycle analysis, propidium iodide (PI)/RNase staining buffer from BD Pharmingen (556463, New Jersey, USA) was used. Cells were then analyzed on a CytoFlex S flow cytometer (Beckman Coulter, CA, USA). Data analysis was performed with the FlowJo software (San Carlos, CA, USA). Apoptosis was measured with the Annexin V/PI apoptosis detection kit (BD Pharmingen, USA). Cells positive for Annexin V were determined to be apoptotic (28), and were located in the right quadrant of the dot plot. Statistical analysis was performed by ANOVA. P-values below 0.05 in comparison to the control group were considered significant.

### Western Blot

Protein extraction, separation and immunoblotting were performed as previously described (29). The following antibodies were used: anti-PDK1 (CA3062, 1:1000, Cell Signaling Technology, MA, USA), anti-P-PDK1 (Ser241) (CA3061, 1:1000, CST), anti-Akt (CA9272, 1:1000, CST), anti-P-Akt (Ser473) (CA4060, 1:1000, CST), anti-P-Akt (Thr308) (CA9275, 1:1000, CST), anti-CDK2 (CA2546, 1:1000, CST), anti-P-CDK2 (Thr160) (CA2561, 1:1000, CST), anti-PARP (CA9532, 1:1000, CST), anti-Cleaved PARP (CA5625, 1:1000, CST), anti-Caspase-3 (CA9662, 1:1000, CST), anti-Cleaved Caspase-3 (CA9661, 1:1000, CST), anti-P27 (CA3698, 1:1000, CST), anti-HDAC1 (CA5356, 1:1000, CST), anti-HDAC2 (CA5113, 1:1000, CST), anti-HDAC3 (CA3949, 1:1000, CST), anti-HDAC10 (ab108934, 1:1000, Abcam, Cambridge, UK), anti-Histone H3 (CA4499, 1:2000, CST), anti-Histone H3/acetyl K27 (ab4729, 1:1000, Abcam), and secondary HRP-linked antibodies (1:2000, Cell Signaling Technology, MA, USA). Anti-GAPDH (CA60004-1-Ig, 1:10000, Proteintech, Suite, USA) was used as a loading control. Blots were then detected using the hypersensitive ECL chemiluminescence kit (NCM Biotech, Suzhou, China) and the Bio-Rad ChemiDoc XRS + detection system (Bio-Rad, CA, USA).

### RNA-Sequencing

DOHH2 cells were incubated with chidamide for 24h, followed by total RNA extraction with TRIzol reagent (Invitrogen, NY, USA) according to the manufacturer’s instructions. Totally, 1μg total RNA with RIN above 6.5 was used for subsequent library preparation. Next generation sequencing libraries were constructed according to the manufacturer’s protocol. Then, libraries with different indices were multiplexed and loaded on an Illumina HiSeq instrument according to the manufacturer’s instructions (Illumina, CA, USA). Sequencing was carried out using the 2x150 bp paired-end (PE) configuration; image analysis and base calling were conducted with HiSeq Control Software (HCS) + OLB + GAPipeline-1.6 (Illumina) on the HiSeq instrument. KEGG pathway and GO analyses were performed using the R Studio approach. Differential expression analysis used the DESeq2 Bioconductor package, a model based on negative binomial distribution; adjusted p value (padj)<0.05 indicated differential expression.

### *In vivo* Experiments

All animal procedures were performed in accordance with the guidelines of the Animal Care and Use Committee and Ethics Committee of Xiamen University. DOHH2 cells (200μl of PBS, 1×10^7^ cells/mouse) were inoculated subcutaneously into the back of female CB17/Icr-Prkdcscid/IcrlcoCrl mice (approximately 14-16g of body weight, Xiamen University Laboratory Animal Center, Fujian, China). After 3 days, mice were randomly divided into two groups (8 animals per group), to receive vehicle (PBS with 0.2% methyl cellulose/0.1% Tween 80) and chidamide (10 mg/kg/d), administered by oral gavage for 3 successive weeks, respectively. Tumor size and body weight were measured every two days. Tumor volumes were calculated according to the formula V= (L × W^2^)/2 [V, volume (mm3); L, length (mm); W, width (mm)]. After euthanasia, tumor tissues were extracted and divided into two parts: one part was frozen at -80°C for protein extraction followed by Western blot, and the other was fixed with 4% paraformaldehyde for hematoxylin and eosin (H&E) staining, immunohistochemistry (IHC) and immunofluorescence (IF). The slides were incubated overnight at 4°C with primary antibodies targeting Ki67 (27309-1-AP, 1:2000, Proteintech, Suite, USA) and PCNA (10205-2-AP, 1:200, Proteintech) antibodies. Subsequently, DAB (DAB-2032, MXB Biotechnologies, Fujian, China) was applied for 5 min at room temperature according to the manufacturer’s instructions. TUNEL-FITC (A111-03, Vazyme Biotech, Jiangsu, China) was applied at room temperature for 20 min, and analysis was performed under a fluorescence microscope (Nikon, Eclipse Ci-L, Japan).

### Statistical Analyses

Statistical analyses were performed with Statistical Product and Service Solutions (SPSS) 21.0 (IBM Corp., Amronk, New York, USA), GraphPad Prism 6 (GraphPad Software, CA, USA) and Microsoft Office Excel (WA, USA). Unpaired Student’s t-test was performed to compare group pairs. Multiple groups were compared by one-way ANOVA, followed by *post-hoc* Bonferroni test. All quantifications were performed based on at least three independent experiments. P<0.05 was considered statistically significant.

## Results

### Effect of Chidamide on t-FL Cell Viability

**Figure 1:**
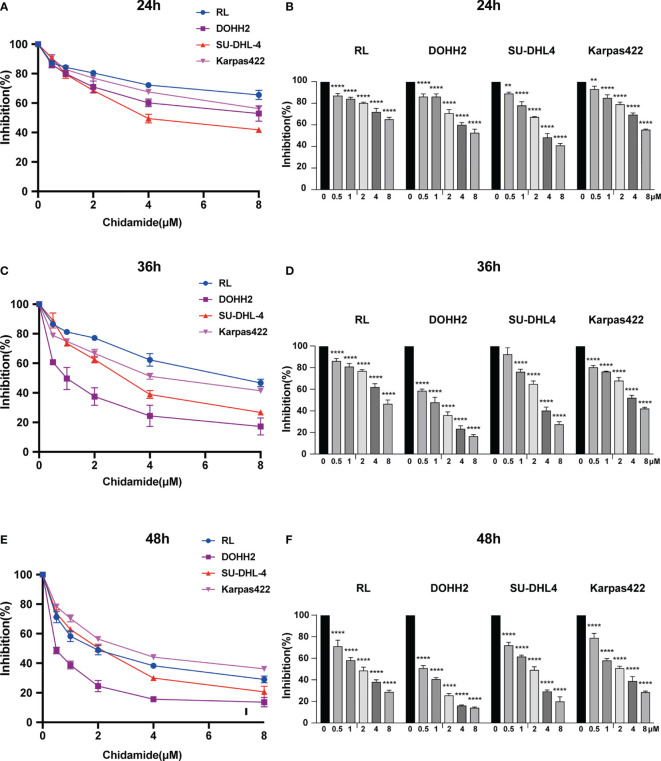
Chidamide inhibits viability in FL cell lines. Human FL RL, DOHH2, SU-DHL4 and Karpas422 cells were exposed to the indicated concentrations of chidamide for **(A)** 24 h, **(C)** 36 h or **(E)** 48 h, after which cell proliferation was measured by the CCK-8 assay. Data (mean ± S.D.) from at least three independent experiments are shown in **(B)** 24 h, **(D)** 36 h and **(F)** 48 h. (**p < 0.01; ****p < 0.0001).

We evaluated the anti-proliferative activity of chidamide in four t-FL cell lines, including RL, DOHH2, SU-DHL4 and Karpas422 cells. As assessed by the CCK-8 assay, after exposure to a series of concentrations for 24, 36 and 48 h, chidamide potently reduced cell viability in all four FL cell lines in a dose-dependent manner (**Figure 1**). The IC_50_ values of chidamide for these four cell lines over different treatment periods (**Supplementary Table 1**) revealed that chidamide inhibition of RL, DOHH2, SU-DHL4 and Karpas422 cells was time-dependent. IC_50_ values for chidamide-treated DOHH2 cells (9.08± 2.03, 0.85 ± 0.07 and 0.54 ± 0.05 μM, respectively) were close to those obtained in SU-DHL4 cells (4.56± 0.31, 3.17± 0.2 and 1.67± 0.05 μM, respectively) following incubation times of 24, 36 and 48 h. However, for the same incubation times, IC_50_ values in RL (30.39± 26.45, 7.447 ± 0.87 and 1.87± 0.25 μM, respectively) and Karpas-422 cells 10.92 ± 0.15, 5.10 ± 0.23 and 3.09 ± 0.23 μM, respectively) decreased steeply with increasing drug exposure time (about 16.0 and 3.0-fold from 24 h to 48 h, respectively), and were higher than the corresponding IC_50_ values recorded for DOHH2 and SU-DHL4 cells. These findings suggested that DOHH2 and SU-DHL4 cells may be more sensitive to chidamide in terms of viability and proliferation.

### Chidamide Induces Caspase-Dependent Apoptosis in t-FL Cells

**Figure 2:**
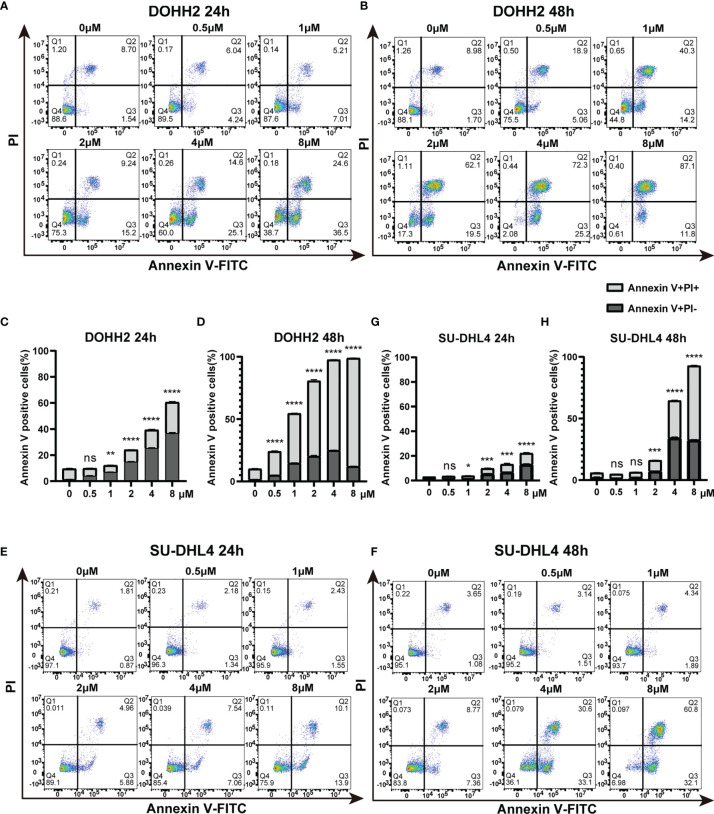
Chidamide induces apoptosis in highly sensitive FL DOHH2 and SU-DHL4 cells. **(A, B)** DOHH2 and **(E, F)** SU-DHL4 cells were treated with various doses of chidamide for 24 h or 48 h, after which the percentages of apoptotic cells were examined by Annexin V/PI double staining. The levels of apoptotic cells were remarkably increased at the indicated times of exposure to chidamide in **(C, D)** DOHH2 and **(G, H)** SU-DHL4 cells. Data are mean ± S.D. (*p < 0.05; **p < 0.01; ***p < 0.001; ****p < 0.0001; NS, *p* > 0.05).

**Figure 3:**
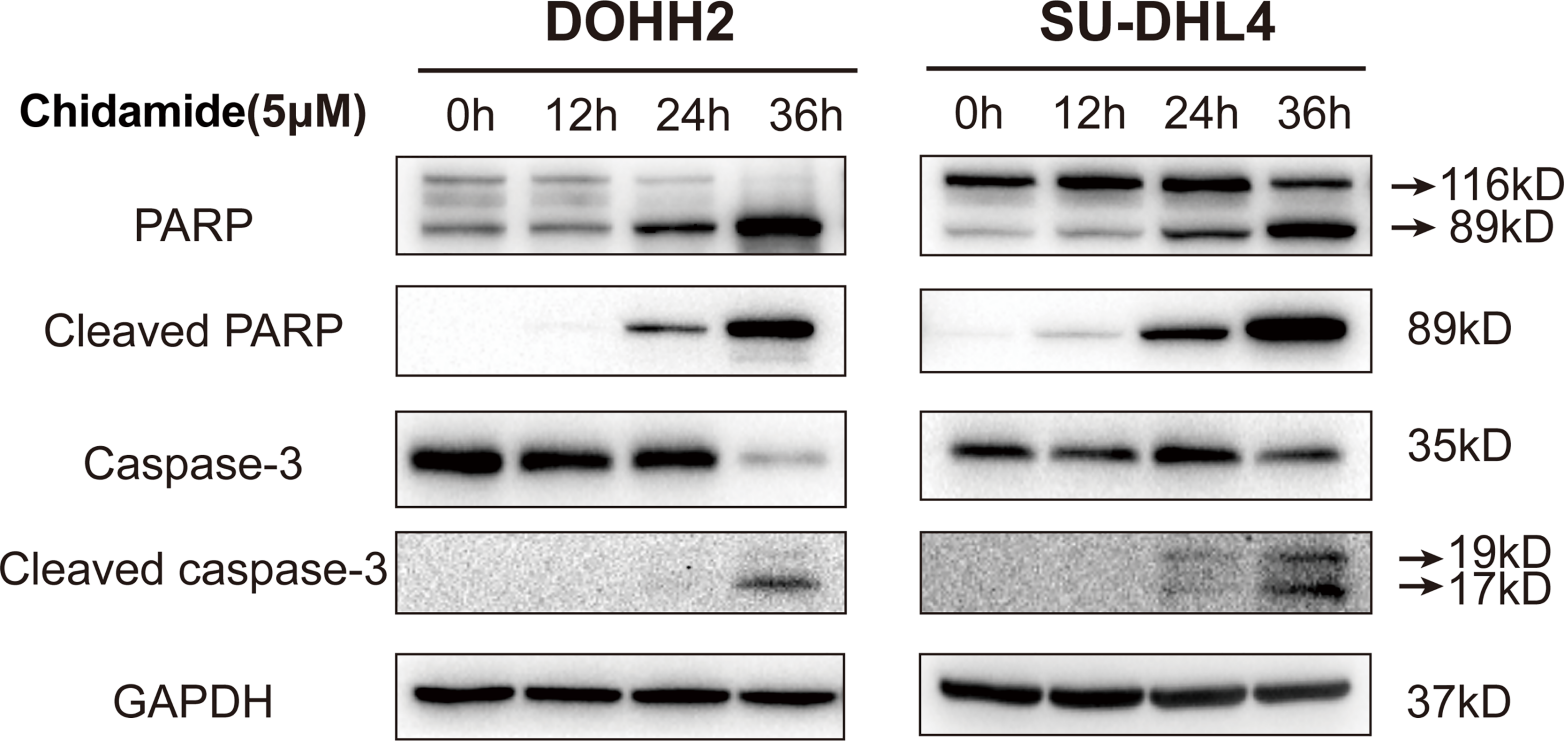
Chidamide induces apoptosis in FL cells by triggering the caspase dependent pathway. Western blot of apoptosis signaling proteins in DOHH2 and SU-DHL4 cells, including PARP, cleaved-PARP, caspase-3 and cleaved caspase-3, exposed to 5μM chidamide for 12h, 24 h and 36 h.

To further assess the antitumor effect of chidamide on t-FL cells, flow cytometry after Annexin V/PI staining was performed to examine whether chidamide induces apoptosis in t-FL cells. Four t-FL cell lines were cultured with increasing concentrations of chidamide for 24 and 48 h before apoptosis assessment. Consistent with the CCK-8 assay, dose and time-dependent induction of cell death was evident in both DOHH2 and SU-DHL4 cells, whereas RL and Karpars422 cells showed reduced, although significant induction of apoptosis (**Figure 2** and **Supplementary Figure 1**), further indicating that DOHH2 and SU-DHL4 cells were more sensitive to chidamide and suggesting cell line-specific differences. Thus, besides the reduced cell proliferation, increased cell death might be another factor contributing to chidamide activity in t-FL cells. We next sought to confirm the potential mechanism underlying chidamide’s anti-apoptotic effect. Western blot was carried out to detect the expression of activated caspase-3 and cleaved PARP after chidamide treatment for 12, 24 and 36 h, respectively, in DOHH2 and SU-DHL4 cells. Chidamide treatment for 24 h markedly upregulated cleaved caspase-3 and cleaved PARP (**Figure 3**). Taken together, these results suggested that chidamide induced apoptosis in FL cells by triggering the caspase dependent pathway.

### Chidamide Induces Cell Cycle Arrest in the G0/G1 Phase

**Figure 4:**
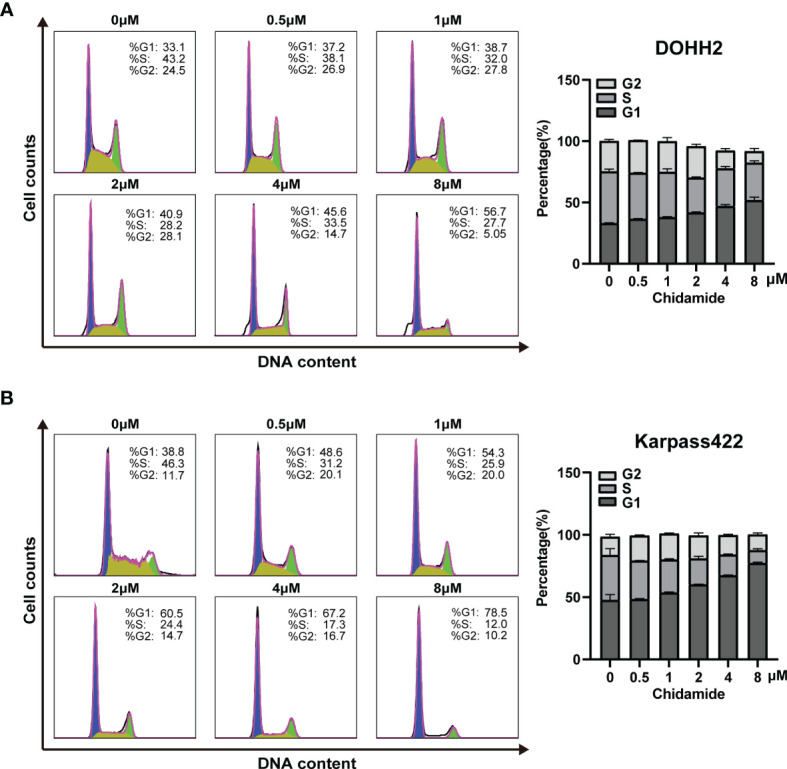
Chidamide induces FL cell cycle arrest in the G0/G1 phase. DOHH2 and Karpas422 cells were treated with chidamide for 24 h at the indicated concentrations, and cell cycle distribution in **(A)** DOHH2 and **(B)** Karpas422 cells was analyzed by flow cytometry. Representative flow cytograms are shown in the right panel.

Aberrant HDAC expression has previously been shown to impair a subset of genes involved in cell cycle regulation. Accordingly, inducing cell cycle arrest may be an underlying mechanism of chidamide’s effect on FL cells. In the above CCK-8 assay, chidamide exhibited a potent inhibitory effect on the proliferation of all four FL cells studied. We further investigated the effect of chidamide on cell cycle distribution in these four FL cell lines. After 24 h of incubation with various concentrations of chidamide, cell cycle analysis by flow cytometry revealed that chidamide induced the accumulation of cells in the sub-G0/G1 phase and reduced cells in the S phase in a concentration dependent manner, with little change in the G2 phase in RL, DOHH2, SU-DHL4 and Karpas422 cells (**Figure 4** and **Supplementary Figure 2**). These data indicated that chidamide inhibited the proliferation of FL cells by inducing cell cycle arrest.

### Transcriptional Signature of Chidamide’s Effects in t-FL Cells

**Figure 5:**
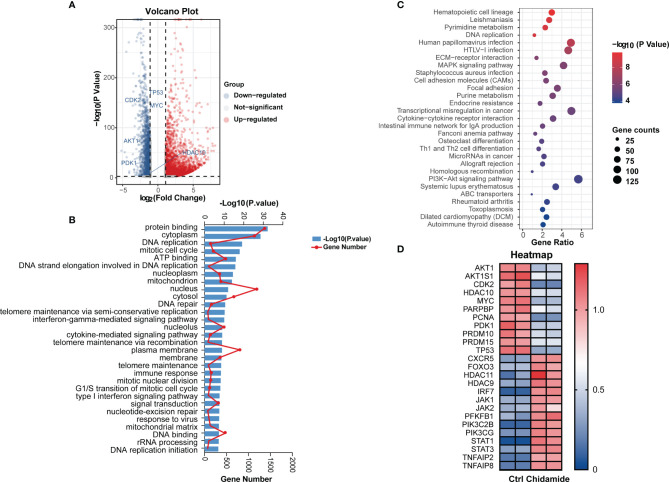
Effects of chidamide in genome-wide gene expression and relative pathway in DOHH2 cells. RNA-seq was performed to profile genome-wide gene expression in DOHH2 cells treated with 5μM chidamide for 24 h. **(A)** Volcano plot depicting 4114 and 2095 genes significantly upregulated and downregulated compared with the DMSO control groups, respectively. **(B)** Gene Ontology (GO) enrichment analysis of DEGs was performed by Gene Set Enrichment Analysis (GSEA). Pathways affected by chidamide in the DOHH2 cell line. **(C)** Differential signaling pathways in KEGG pathway enrichment analysis showing genes involved in PI3K/AKT signaling were significantly enriched. **(D)** Heat map of differentially expressed genes in DOHH2 cells in response to chidamide for 24 h.

**Figure 6:**
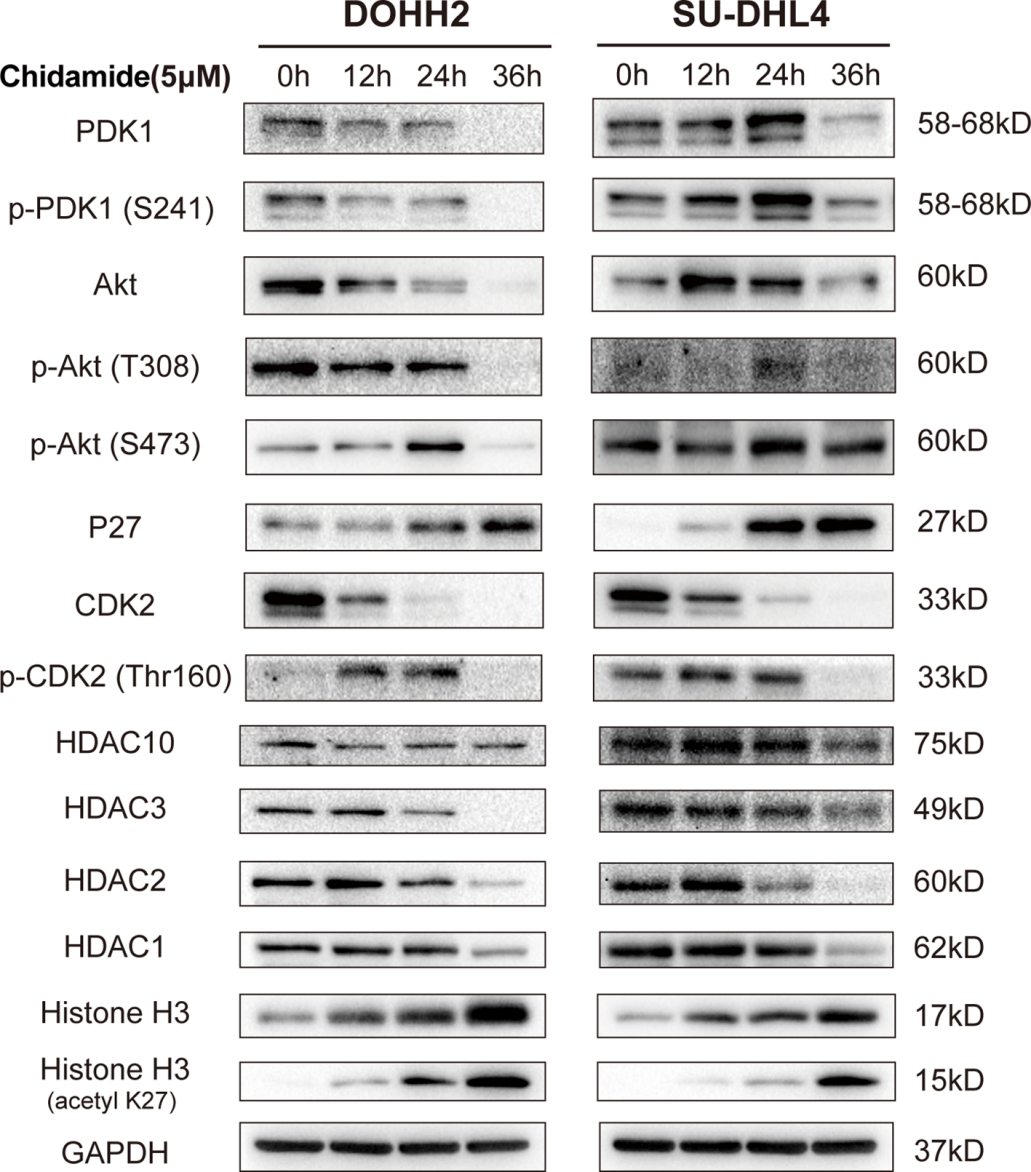
Differential gene and protein expression in FL cells after chidamide treatment. DOHH2 and SU-DHL4 cells were treated with 5μM chidamide for the indicated times. The levels of differential genes were determined by Western blot.

To obtain a global profile of the transcriptional changes after chidamide treatment, we performed genome-wide gene expression (GEP) on the most sensitive cell line (DOHH2 cells) treated with DMSO or chidamide (5µM) for 24 h. Totally, 4114 and 2095 genes in DOHH2 cells were significantly upregulated and downregulated (log2FC≥1, P<0.05) by chidamide, respectively (**Figure 5A**). KEGG analysis revealed that chidamide affected several important biological processes, including DNA replication, MAPK signaling, PI3K/AKT signaling and cell cycle regulation (**Figures 5B, C**). The downregulated transcripts mainly comprised HDAC, P53 and CDK2 or genes involved in the PI3K/AKT pathway (**Figure 5D**). In this context, Western blot was performed to validate the target specificity of chidamide on HDAC1, 2, 3 and 10. As shown in **Figure 6**, exposure of DOHH2 and SU-DHL4 cells to chidamide resulted in time-dependent downregulation of HDAC1, 2, 3 and 10, thereby causing hyper-acetylation of histones H3 and H4. Chidamide also upregulated p27 and downregulated phosph-CDK2 (Thr160) in a time-dependent manner. However, Western blot detected no expression change for P27 in RL and Karpas-422 cells (**Supplementary Figure 3**), which might be partially responsible for their lower sensitivity to chidamide. Taken together, these results suggested that the mechanism underlying the anti-proliferative activity of chidamide might involve downregulation of molecules related to the PI3K kinase pathway and cell cycle arrest in t-FL cells.

### Chidamide Affects the PI3K/PDK1/AKT Signaling Pathway in t-FL Cells

Chidamide negatively regulated transcripts encoding MYC- and P53-regulated genes, as well as PI3K/AKT signaling pathway effectors, including HDAC10, AKT1, CDK2, MYC, PARPBP, PCNA, PDK1, PRDM10 and PRDM15 in DOHH2 cells (**Figure 5D**). The inhibitory effect of chidamide on the PI3K/AKT signaling pathway was further confirmed at the protein level. Western blot showed a marked reduction in the expression of PDK1 and phospho-AKT (Ser473 or Thr308) in both DOHH2 and SU-DHL4 cells after chidamide treatment for 36 h (**Figure 6**). In contrast, chidamide did not affect the expression of phospho-AKT (Ser473) in RL and Karpas-422 cells (**Supplementary Figure 3**). Thus, we speculated that chidamide might promote apoptosis and suppress proliferation in part by disrupting the PI3K/PDK1/AKT signaling pathway in t-FL cells.

### Chidamide Has Antitumor Activity in a FL Tumor Xenograft Model

**Figure 7:**
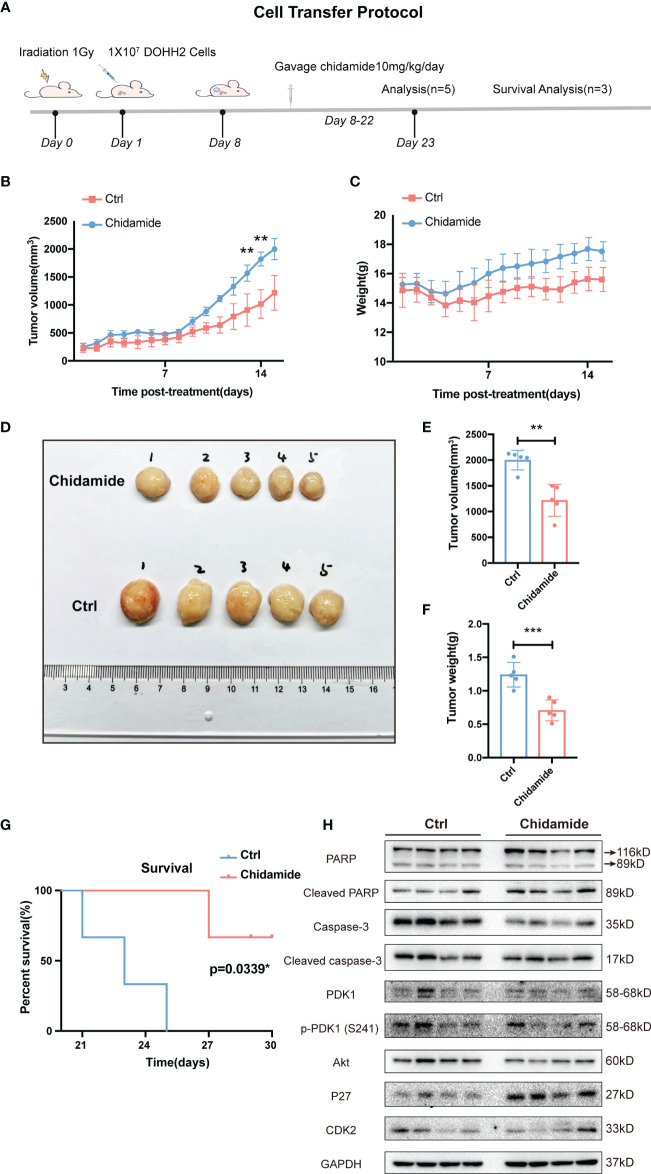
Effect of chidamide on tumor growth in xenograft mouse models. **(A)** Cell injection protocol in a FL tumor xenograft model. Tumor volumes **(B)** and body weights **(C)** of mice were measured daily and presented as mean ± S.D. **(D)** Images of tumors from DOHH2-bearing xenograft mice after the indicated treatments (n=10). Tumor volumes **(E)** and weights **(F)** in the control and chidamide groups were compared to evaluate the treatment response to chidamide. **(G)** Kaplan Meier overall survival (OS) curves of tumor-bearing xenograft mice. **(H)** Chidamide suppressed the PDK1-Akt-P27-CDK2 signaling pathway *in vivo*. The protein levels of PDK1, AKT, P27, CDK2, PARP, cleaved-PARP, caspase3 and cleaved-caspase-3 were determined by Western blot. (**p < 0.01; ***p < 0.001).

Finally, the anti-tumor activity of chidamide was examined in a CB17/Icr-Prkdcscid/IcrlcoCrl mouse xenograft model bearing DOHH2 cells. In this study, DOHH2 cells (10^7^) were injected subcutaneously into the back of mice, which were randomly divided into the vehicle control and chidamide groups. Vehicle or chidamide (10 mg/kg/day) was orally given continuously daily for three weeks (**Figure 7A**). After chidamide treatment for 13 days, compared with the control group, mouse tumors showed obvious growth inhibition with no fatal toxicity (**Figures 7B, C**). Although a temporary body weight loss was observed at the beginning of chidamide administration, it was recovered after a short period of time. Tumors were collected from 5 mice randomly selected per group at the study endpoint (Day 22). As shown in **Figure 7D**, chidamide treatment resulted in a marked reduction of tumor burden, reflected by decreased volume and weight of tumor masses, compared with the vehicle control (**Figures 7E, F**). Besides, chidamide significantly prolonged survival in the treatment group compared with vehicle treated animals (**Figure 7G**).

**Figure 8:**
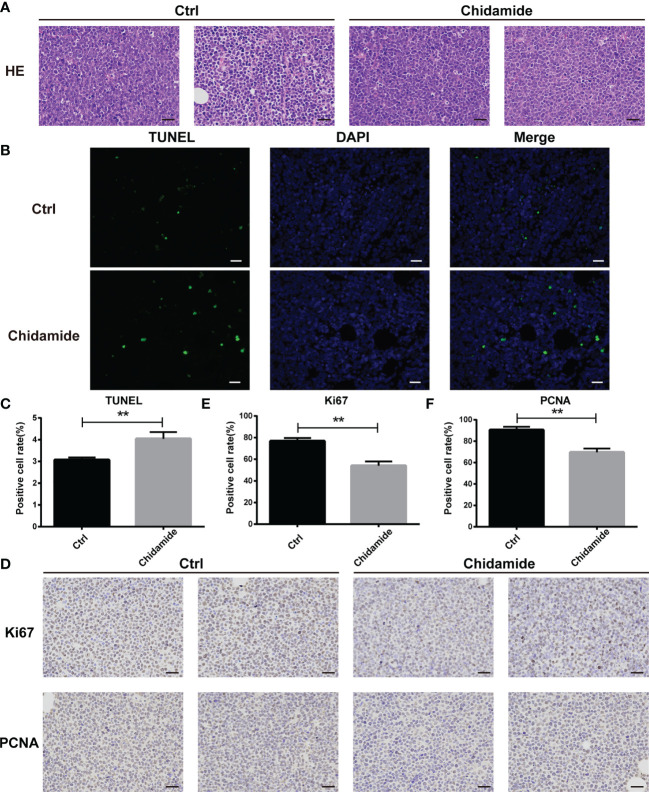
Inhibition of tumor growth *in vivo* by chidamide. **(A)** Tumor samples collected after treatment with vehicle/control or chidamide were fixed, sliced and stained with H&E. **(B)** Representative images of immunofluorescent TUNEL staining performed on serial sections of the tumors are shown; the corresponding statistical results are shown in **(C)**. Images were acquired under a Nikon microscope (original magnification, ×400). **(D)** The expression levels of Ki67 and PCNA were detected by immunohistochemical staining. **(E, F)** Data represent three independent experiments, and are mean ± S.D. (**p < 0.01).

The tumor tissues obtained from chidamide treated animals displayed obvious nuclear shrinkage as shown by H&E staining (**Figure 8A**). Apoptosis in tumor tissues was detected by the TUNEL assay. The number of apoptotic cells was markedly increased in chidamide treated tumors (**Figures 8B, C**), in agreement with *in vitro* findings. Immunohistochemical staining revealed that Ki-67 and PCNA levels were decreased in tumor tissues from chidamide treated mice (**Figures 8D–F**). Compared with the vehicle control group, chidamide treated mice showed significantly reduced p-PDK1 expression, and markedly increased expression of P27, cleaved caspase-3 and cleaved PARP (**Figure 7H**). These results were consistent with *in vitro* findings. Taken together, these data suggested that chidamide effectively inhibited t-FL tumorigenesis and development *in vivo*.

## Discussion

Transformed follicular lymphoma (t-FL) is considered a disease predominantly caused by several epigenetic aberrations (e.g., mutations affecting the epigenetic modifiers KMT2D, EZH2, CREBBP and MEF2B) rather than sequential acquisition of genetic aberrations (8, 18). Thus, targeting enzymes involved in the regulation of DNA methylation and histone modifications might be critical for developing more effective treatment strategies in t-FL. In this regard, histone deacetylases (HDACs) represent the most widely studied therapeutic targets, with HDAC inhibitors (HDACi) including chidamide, vorinostat and romidepsin, approved for use in the therapy of cutaneous T-cell lymphoma (23, 30). However, clinical practice has not yet delivered desirable results by applying HDACi in the treatment of t-FL. Furthermore, the biological effects of HDACi in t-FL remain unclear, as well as the identification of response mechanisms. Taking these observations into consideration, we evaluated the activity and underlying mechanism of a benzamide-type selective HDAC inhibitor, chidamide, in preclinical models of t-FL cells.

HDACs have been demonstrated to play a crucial role in the pathogenesis of lymphoma (31, 32). Gil et al. reported that aberrant expression of HDAC9 could lead to lymphoproliferative disorders, including germinal center (GC) and post-GC lymphomas (33). In follicular lymphoma (FL) and diffuse large B cell lymphoma (DLBCL), Bcl6 recruits HDAC3 to repress transcription and trigger B cell lymphoma (34). All these data strongly suggest that HDACs are promising therapeutic targets for GC lymphomas. Furthermore, HDAC inhibitors have been reported to induce Bcl6 downregulation in GC lymphomas including DLBCL (35). Herein, we demonstrated that chidamide had an anticancer effect as a single agent in several t-FL cell lines. At clinically achievable concentrations, chidamide showed cell type- and dose-dependent cytotoxicity in t-FL cells at 24 h, with IC50 values ranging from 4.5µM to 30µM, and varied responses to chidamide among different cells. In DOHH2 and SU-DHL4 cells, which were relatively more sensitive to chidamide, a higher degree of apoptosis induced by chidamide was found as strongly evidenced by the activation of caspase-3. This is likely due to the cell origins or unknown differences of their genetic differentiation.

Since chidamide inhibited cell proliferation and induced apoptosis in t-FL cells, we further investigated whether chidamide regulates cell cycle progression, which is one of the main mechanisms by which HDACi induce tumor cell death. In this context, previous studies have shown that G1 arrest appears to be a common response to chidamide in various tumor cells (36–38). Thus, cell cycle regulators, including cyclins and CDK inhibitors (e.g., p21 and p27), may be tightly controlled by chidamide (27, 36, 39). In general, p27 is known to control G1 length and cell cycle exit by inhibiting the kinase activity of CDK2 bound to cyclin E, thereby causing the dephosphorylation of retinoblastoma protein (Rb), which blocks E2F activity in the transcription of genes required for G1/S transition (40, 41). In this study, microarray analysis of chidamide-treated t-FL cells pointed to cell cycle arrest in the G1 phase. Upon chidamide treatment, CDK2 was found to be specifically inhibited in DOHH2 and SU-DHL4 cells accompanied by p27 activation. However, obvious changes of p27 expression were not observed in RL and Karpas-422 cells, in disagreement with the anti-proliferative phenotype as well as G1 arrest in these cells, indicating that p27-mediated inhibition of CDK2 might partially contribute to chidamide-induced cell cycle arrest. On the other hand, we showed that chidamide treatment simultaneously caused the accumulation of histone H3 acetylation and the activation of p27 and cell cycle exit in DOHH2 and SU-DHL4 cells. The present findings indicate that the mechanism by which chidamide induces G1 arrest by inhibiting HDACs is cell-specific.

**Figure 9:**
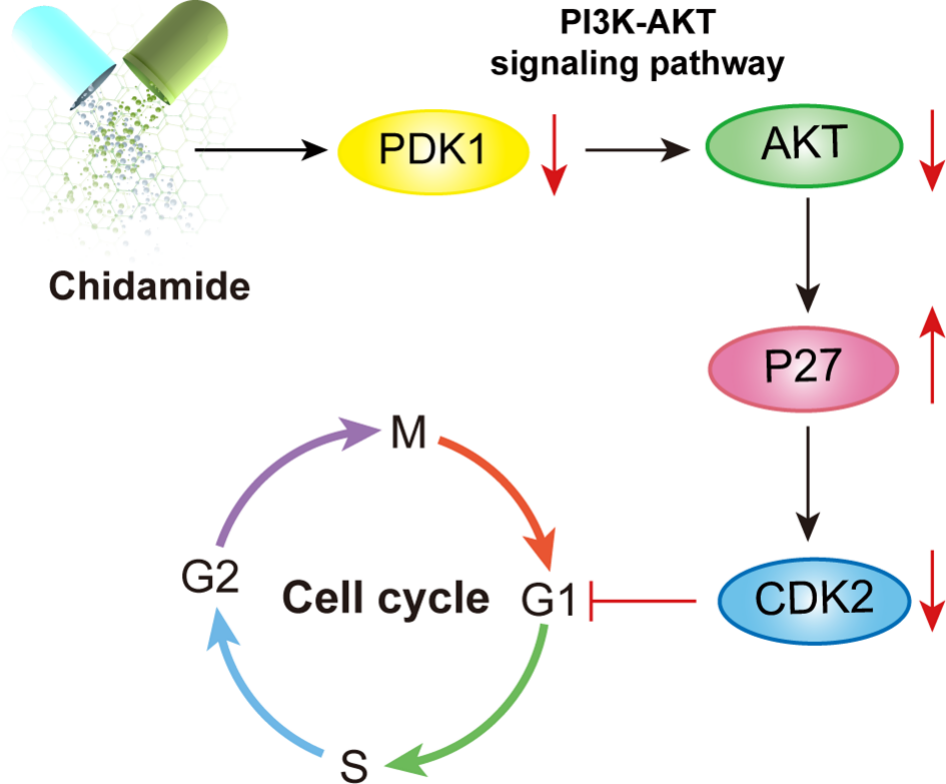
Illustration of the mechanism underlying chidamide induced follicular lymphoma growth inhibition. Chidamide exerts antitumor activity toward FL both *in vitro* and *in vivo*, potentially by targeting the PDK1-Akt-P27-CDK2 pathway and triggering caspase dependent apoptosis, finally markedly blocking cells in the G0/G1 phase.

The integration of gene expression profiling and sensitivity in cancer cells allowed the identification of functional pathways that might predict the response to chidamide (27, 42). DOHH2 cells with the highest sensitivity to chidamide had high expression levels of genes involved in the PI3K/PDK1/AKT pathway, which is regulated in several human carcinomas, including lymphoid malignancies (43, 44). In addition, an important biological effect of chidamide, both *in vitro* and *in vivo*, was the downregulation of the PI3K/AKT pathway. Several studies have indicated that PI3K/AKT pathway downregulation is a relevant mechanism of chidamide’s effects in various cancer cell lines (27, 45, 46). The PI3K/AKT pathway was shown to be activated in human cancers by oncogenic mutations of the PIK3CA gene encoding the catalytic subunit p110a (47). In lymphoid malignancies, including DLBCL (48), MCL (49) and FL (50), hyperactivation of AKT is due to its enhanced activation/phosphorylation at Serine 473 (Ser473). In accordance with apoptosis and caspase-3 activation observed at 24 to 48 h, AKT phosphorylation at ser473 was inhibited in both DOHH2 and SU-DHL4 cells by chidamide treatment. Taken together, these findings suggested that chidamide modulated PI3K/AKT signaling, which is known to be involved in cell proliferation, cell cycle regulation, apoptosis and tumor development (**Figure 9**).

As the activity of chidamide, as monotherapy at the doses tested in this study was modest, understanding potential biomarkers that are predictive of response is very important for the design of future clinical trials (51). In addition, emerging data from *in vitro* studies indicate that HDAC inhibitors, such as chidamide, may have improved activity when used in combination therapy (52–54). To this end, several recent studies have provided a strong preclinical rationale for combination with chemotherapy, immunotherapy, or molecular targeted therapy, paving the way for possible studies in selected populations (53–55). In our previous work, we revealed that ABT-199 (25) or MLL-menin inhibitor (56) has a synergistic inhibitory effect on acute myeloid leukemia cells when combined with chidamide. Combination with a demethylating agent also showed benefit in diffuse large B cell lymphomas (DLBCLs) (53). In addition, chidamide could increase PD-L1 expression in the tumor microenvironment, and preclinical studies have demonstrated synergy between chidamide, and PD-1 blockade in solid tumors (55, 57). A single-arm-phase II study is therefore currently ongoing to evaluate the activity of chidamide in combination with sintilimab in relapsed or refractory peripheral T-cell lymphomas (58). It is possible that chidamide administration in the context of these combination strategies could further enhance the killing of tumor cells.

In summary, this study demonstrated the importance of HDACs in the progression and transformation of FL, and provided a critical link between epigenetic changes and increased FL aggressiveness. We also provided evidence that chidamide exerts anticancer effects by inducing G1 arrest and apoptosis *via* PI3K/PDK1/AKT signaling pathway inactivation. Furthermore, in t-FL cells relatively sensitive to chidamide-induced apoptosis, chidamide caused significant changes in the transcriptome profile, providing a compelling rationale for chidamide as an effective single-agent in aggressive FL. The data presented here provide the basis for further exploration of chidamide in combination therapies.

## Data Availability Statement

The datasets presented in this study can be found in online repositories. The names of the repository/repositories and accession number(s) can be found below: SAMN22127095.

## Ethics Statement

The animal study was reviewed and approved by the Animal Care and Use Committee and Ethics Committee of Xiamen University. Written informed consent was obtained from the owners for the participation of their animals in this study.

## Author Contributions

Conception and design: BX, JZ, and MZ. Development of methodology: MZ, JT, GP, YJ, and HZ. Analysis and interpretation of data: GP, QL, QC, and LF. Technical support: MD, BX, and JZ. Writing, review, and/or revision of the manuscript: JZ and MZ. Study supervision: BX and JZ. All authors contributed to the article and approved the submitted version.

## Funding

This study was supported by the National Natural Scientific Foundation of China (No. 82170180,81770126, 81800163), Fujian Natural Science Foundation of China (No. 2020J011246), Xiamen Municipal Bureau of Science and Technology (No. 3502Z20209003) and Lymphoma Research Fund of Chinese Anti-Cancer Association (No. CORP-117).

## Conflict of Interest:

The authors declare that the research was conducted in the absence of any commercial or financial relationships that could be construed as a potential conflict of interest.

## Publisher’s Note

All claims expressed in this article are solely those of the authors and do not necessarily represent those of their affiliated organizations, or those of the publisher, the editors and the reviewers. Any product that may be evaluated in this article, or claim that may be made by its manufacturer, is not guaranteed or endorsed by the publisher.

## Supplementary Material

Supplementary Figure 1 Chidamide induces apoptosis in relatively *de novo* resistant FL RL and Karpas422 cells. Representative flow cytograms depicting RL **(A, B)** and Karpas422 **(E, F)** cells exposed for 24 h or 48 h to chidamide. Data represent three independent experiments examining RL **(C, D)** and Karpas422 **(G, H)** cells, and are mean ± S.D. (**p* < 0.05; ***p* < 0.01; ****p* < 0.001; *****p* < 0.0001; NS: *p* > 0.05)

Supplementary Figure 2 Chidamide induces G0/G1 arrest in FL cell lines. Representative flow cytograms of SU-DHL4 **(A)** and RL **(B)** cells treated for 24 h with chidamide.

Supplementary Figure 3 Effect of chidamide on protein expression in resistant FL RL and Karpas422 cell lines. RL **(A)** and Karpas422 **(B)** cells were treated with 5μM chidamide for the indicated times. The levels of phospho-AKT (Ser473) and P27 were determined by Western blot.

Supplementary Table 1 IC50 values of chidamide in FL cell lines.

## Abbreviations

t-FL, transformed follicular lymphoma; DLBCL, Diffuse large B-cell lymphoma; HDACs, Histone deacetylases; HATs, histone acetyltransferases; NHL, Non-Hodgkin lymphoma; R/R, Relapsed or refractory; FBS, Fetal bovine serum; CCK-8, Cell Counting Kit-8; qRT-PCR, Real-time quantitative PCR; GO, Gene Ontology; KEGG, Kyoto Encyclopedia of Genes and Genomes; PI, Propidium iodide; SPSS, Statistical Product and Service Solutions; GSEA, gene set enrichment analysis.
