# Supplementary figures and images for: Preclinical Evaluation of the HDAC Inhibitor Chidamide in Transformed Follicular Lymphoma
Source: Front Oncol. 2021 Dec 3;11:780118. doi: 10.3389/fonc.2021.780118 (PMC8677934; doi:10.3389/fonc.2021.780118)

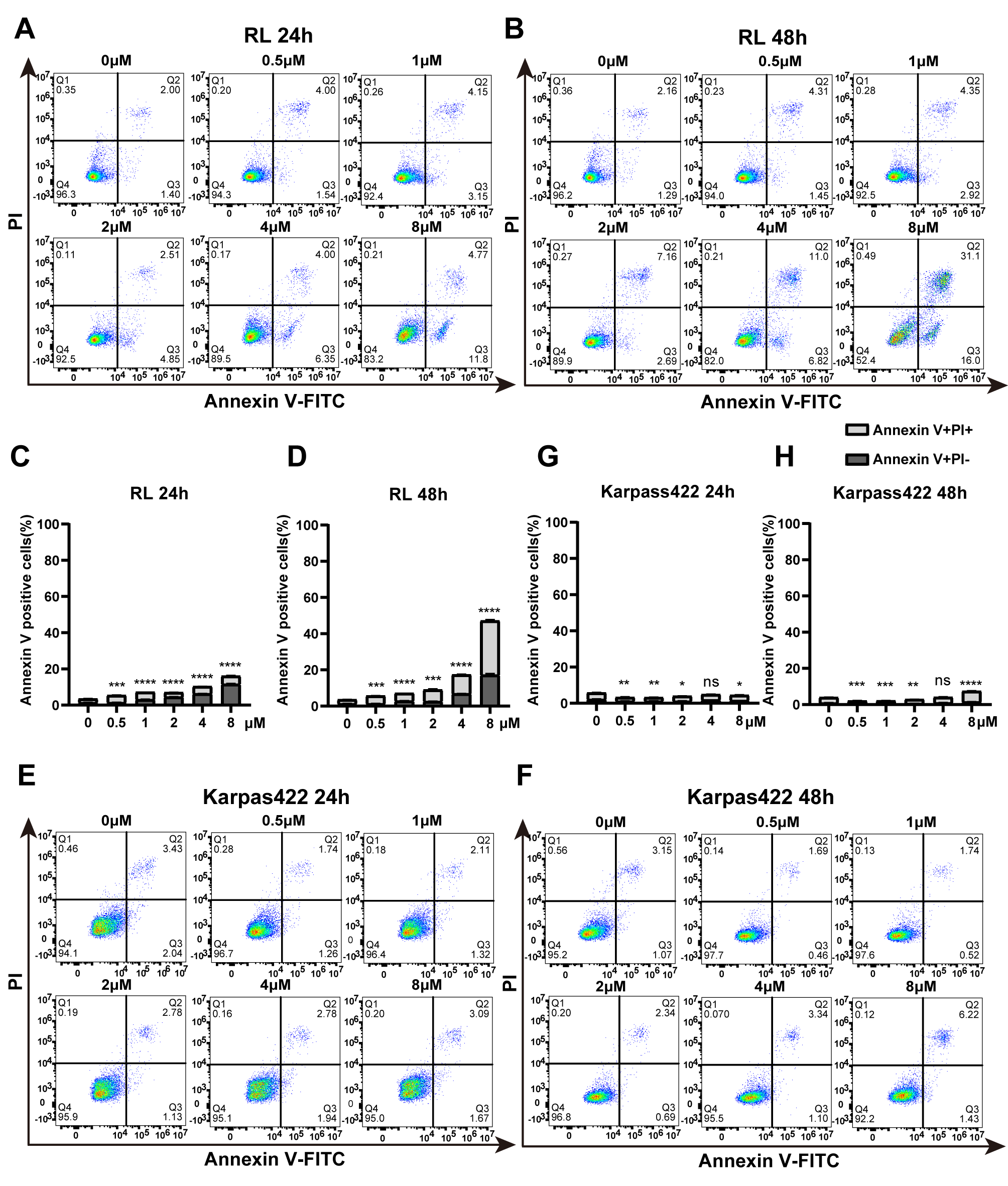

Supplement: Supplementary Figure 1 — Chidamide induces apoptosis in relatively de novo resistant FL RL and Karpas422 cells. Representative flow cytograms depicting RL (A, B) and Karpas422 (E, F) cells exposed for 24 h or 48 h to chidamide. Data represent three independent experiments examining RL (C, D) and Karpas422 (G, H) cells, and are mean ± S.D. (*p < 0.05; **p < 0.01; ***p < 0.001; ****p < 0.0001; NS: p > 0.05) [file Image_1.tif]

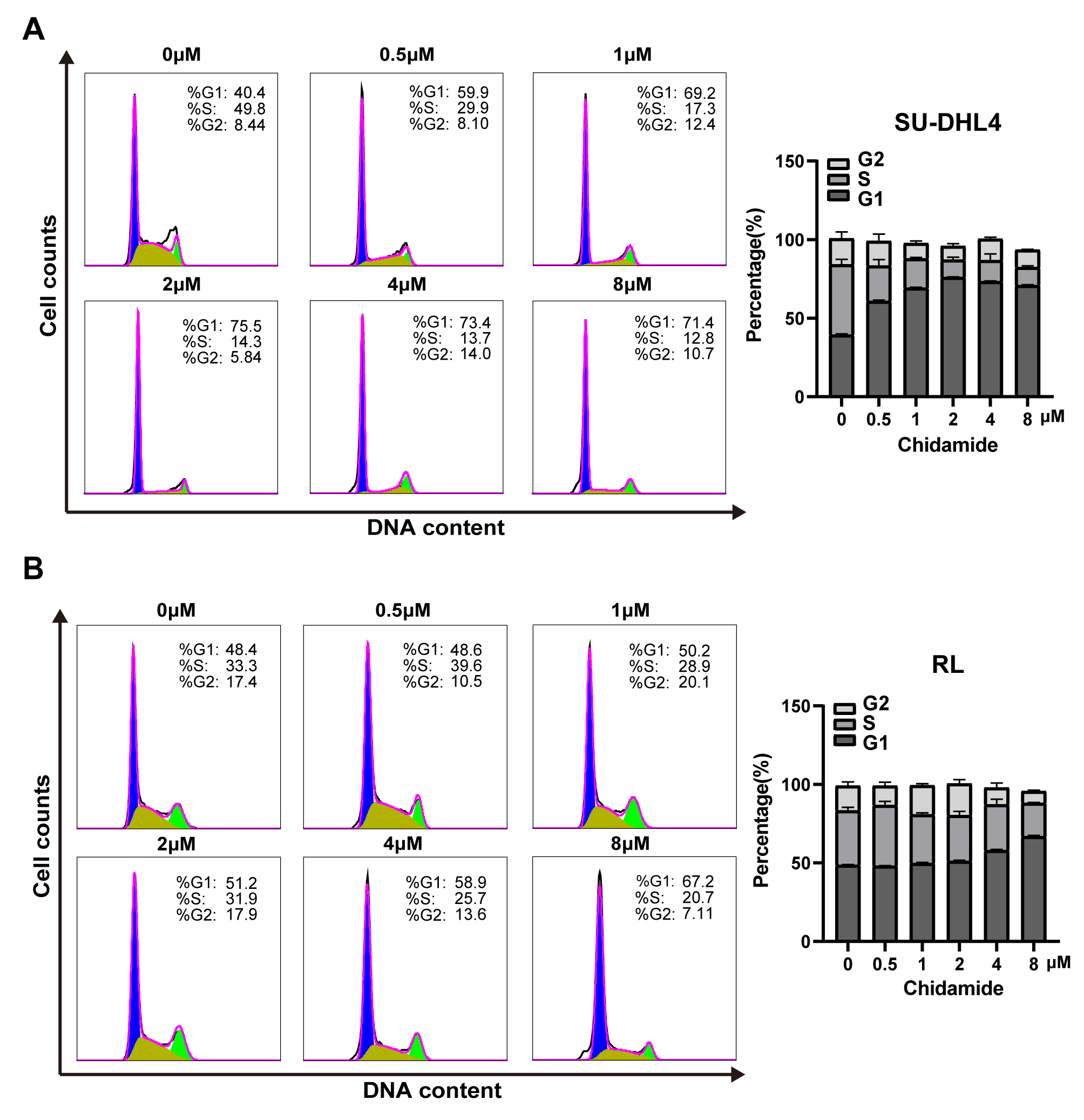

Supplement: Supplementary Figure 2 — Chidamide induces G0/G1 arrest in FL cell lines. Representative flow cytograms of SU-DHL4 (A) and RL (B) cells treated for 24 h with chidamide. [file Image_2.tif]

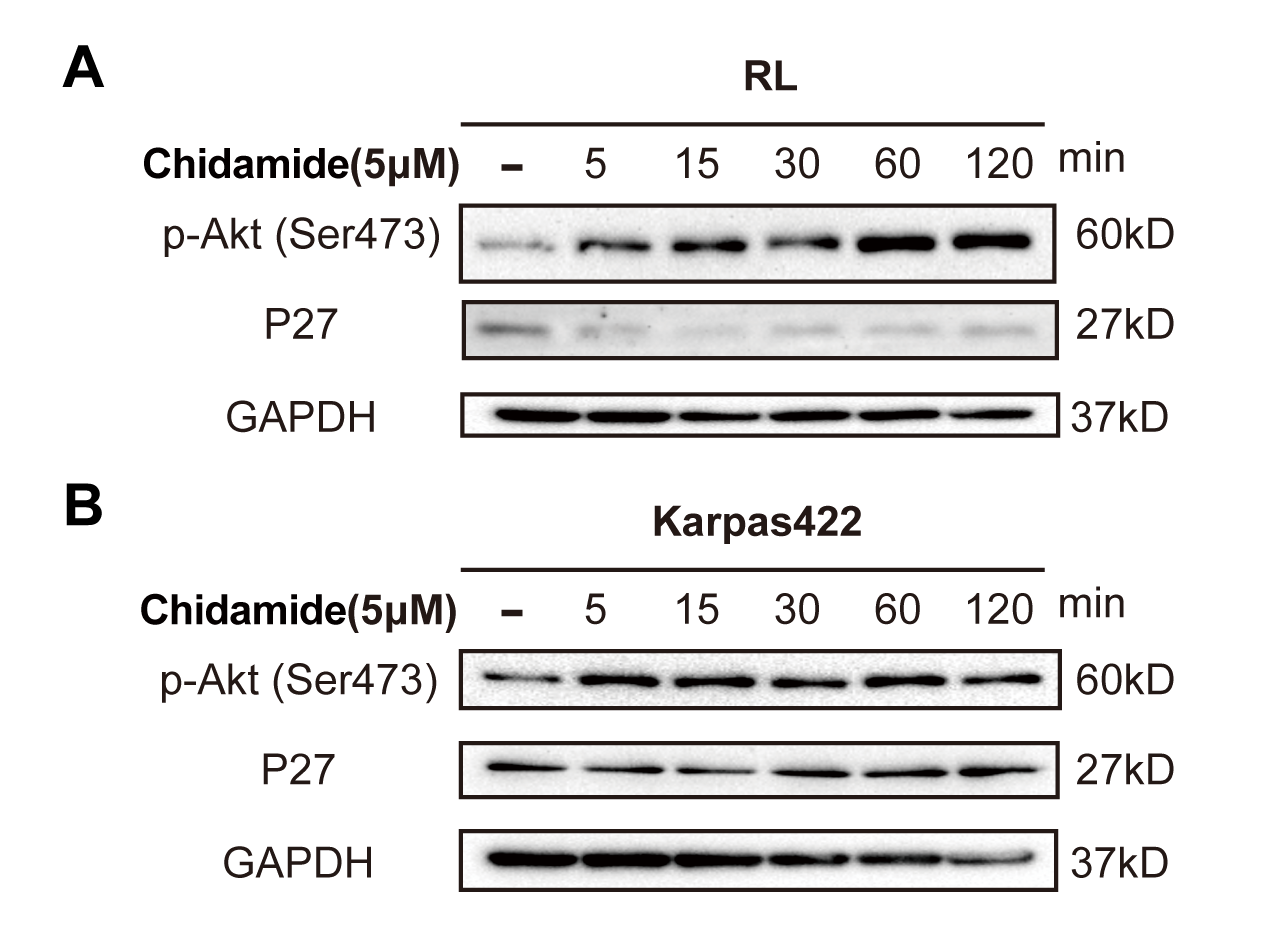

Supplement: Supplementary Figure 3 — Effect of chidamide on protein expression in resistant FL RL and Karpas422 cell lines. RL (A) and Karpas422 (B) cells were treated with 5μM chidamide for the indicated times. The levels of phospho-AKT (Ser473) and P27 were determined by Western blot. [file Image_3.tif]
