# Supplementary material for: Preclinical Evaluation of the HDAC Inhibitor Chidamide in Transformed Follicular Lymphoma
Source: Front Oncol. 2021 Dec 3;11:780118. doi: 10.3389/fonc.2021.780118 (PMC8677934; doi:10.3389/fonc.2021.780118)
Supplement: Supplementary Table 1 — IC50 values of chidamide in FL cell lines. [file Table_1.docx]

**Supplementary Table 1. IC50 values of chidamide in FL cell lines.**

| **FL Cell Line** | IC50 ± S.D (μmol/L) | | |
| --- | --- | --- | --- |
|  | 24 h | 36 h | 48 h |
| **RL** | 30.39± 26.45 | 7.447 ± 0.87 | 1.87± 0.25 |
| **DOHH2** | 9.08± 2.03 | 0.85 ± 0.07 | 0.54 ± 0.05 |
| **SU-DHL4** | 4.56± 0.31 | 3.17± 0.2 | 1.67± 0.05 |
| **Karpas422** | 10.92 ± 0.15 | 5.10 ± 0.23 | 3.09 ± 0.23 |
